# Platinum Nanozyme Probes for Cellular Imaging by Electron Microscopy

**DOI:** 10.1002/smsc.202400085

**Published:** 2024-06-09

**Authors:** Elisa De Luca, Deborah Pedone, Anna Scarsi, Roberto Marotta, Federico Catalano, Doriana Debellis, Lorenzo Cursi, Benedetto Grimaldi, Mauro Moglianetti, Pier Paolo Pompa

**Affiliations:** ^1^ Nanobiointeractions&Nanodiagnostics Istituto Italiano di Tecnologia via Morego 30 16163 Genova Italy; ^2^ Institute of Nanotechnology (NANOTEC) National Research Council Via Monteroni 73100 Lecce Italy; ^3^ Istituto Italiano di Tecnologia Center for Biomolecular Nanotechnologies Via Barsanti 73010 Arnesano, Lecce Italy; ^4^ Electron Microscopy Laboratory Nanochemistry Department Istituto Italiano di Tecnologia Via Morego 30 16163 Genova Italy; ^5^ Molecular Medicine Research Line Istituto Italiano di Tecnologia (IIT) 16163 Genoa Italy; ^6^ Center for Cultural Heritage Technology (CCHT) Istituto Italiano di Tecnologia (IIT) Via Torino 155 30172 Venezia Italy

**Keywords:** cellular imaging, nanozyme probes, platinum nanoparticles, transmission electron microscopy

## Abstract

The highly efficient peroxidase‐like activity of platinum nanozymes (3–20 nm size) is exploited within the complex cellular environment to catalyze the oxidation of the DAB substrate, producing an electron‐dense signal around the nanozyme surface, upon osmium staining. It is proved that such nanozyme amplification can achieve a catalytic signal enhancement up to 10‐fold, enabling the quick detection of the Pt particles (even of 3 nm size) by transmission electron microscopy (TEM) also at low magnification and across wide fields of view in the intricate intracellular milieu. The developed procedure is ideally suited to overcome standard amplification strategies currently used in TEM analysis, such as gold or silver enhancements. Furthermore, the wide versatility of the Pt‐nanozyme probes in TEM imaging is demonstrated in immuno‐EM and protein trafficking studies, showing their potential to track the subcellular localization of target biomolecules at both low and high magnifications. These results suggest that the use of nanozymes might represent a paradigm shift in the conventional amplification systems currently employed in electron microscopy for cellular analyses, offering enhanced imaging capabilities.

## Graphical abstract

This study introduces a novel technique, exploiting platinum nanozyme probes, to improve the localization of specific cellular components via transmission electron microscopy. This approach represents a significant development with respect to traditional amplification systems in electron microscopy, offering advanced imaging capabilities for detailed cellular analyses.

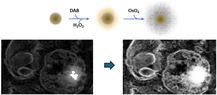

## 1 Introduction

In the latest years, interesting advances in nanomedicine and nanodiagnostics are emerging in academic research thanks to the introduction of nanozymes.^[^ ^1^, ^2^, ^3^, ^4^, ^5^, ^6^, ^7^, ^8^, ^9^, ^10^, ^11^ ^]^ Artificial enzymes, namely catalytic nanomaterials endorsed with enzyme‐like activities, represent a promising alternative to natural enzymes, due to their long‐term stability and versatility.^[^ ^1^ ^]^ Among the vast range of nanomaterials, platinum nanoparticles (PtNPs) are raising increasing interest as efficient enzyme substitutes (type 2 nanozymes),^[^ ^12^ ^]^ showing superior catalytic performances, strong robustness against harsh pH and temperature conditions, and easy and low‐cost production and purification protocols.^[^ ^13^, ^14^, ^15^, ^16^ ^]^ Moreover, their catalytic reactivity can be tailored by controlling the physico‐chemical properties of both the environment and the particles.^[^ ^14^, ^17^ ^]^ Major advancement comes from the ability of these nanocatalysts to mimic antioxidant enzymes within complex systems, such as cellular environments. Notably, it has been demonstrated that PtNPs are quite stable in intracellular milieu^[^ ^18^ ^]^ and exhibit robust and versatile antioxidant characteristics, being able to reduce reactive oxygen species levels,^[^ ^15^, ^19^ ^]^ for example, restoring the cellular physiological homeostasis in oxidative stress‐related disorders.^[^ ^10^ ^]^ These important catalytic properties have been shown also in animal studies in which the level of complexity often poses severe obstacles for nanomaterials. Indeed, in *in vivo* models of photoreceptors affected by age‐related macular degeneration, PtNPs have been proven capable to halt the vicious circle connecting oxidative stress, degeneration, and inflammation thanks to their antioxidant and anti‐inflammatory properties.^[^ ^20^ ^]^

In the diagnostic field, PtNPs have been used to mimic the peroxidase (POD) activity of the most widely employed enzyme horseradish peroxidase (HRP), catalyzing the redox reaction between chromogenic molecules, such as 3,3′,5,5′‐Tetramethylbenzidine (TMB), 3,3′‐Diaminobenzidine (DAB), and similar substrates, and the oxidizing cofactor hydrogen peroxide (H_2_O_2_).^[^ ^14^ ^]^ The large surface‐to‐volume ratio and the excellent POD mimicking activities of PtNPs^[^ ^21^ ^]^ ensure high catalytic performances. Since the first example of a PtNP‐based assay reported in the literature more than 10 years ago,^[^ ^22^ ^]^ several nanosensors have been developed to detect cancer cells, metal ions, and bacteria.^[^ ^14^, ^23^, ^24^ ^]^ Moreover, PtNPs can replace peroxidase enzymes and enable rapid, non‐invasive assessment of the body's total antioxidant capacity via saliva, offering a sensitive detection method using naked‐eye or smartphone‐based inspection.^[^ ^25^ ^]^ Furthermore, also thanks to their easy functionalization and purification procedures, PtNPs have shown to be an ideal platform for the development of naked‐eye immunoassays with enhanced sensitivity and stability. Probes composed of antibodies and PtNPs have been proved as more sensitive detection systems for lateral flow immunoassays,^[^ ^8^, ^26^, ^27^, ^28^, ^29^, ^30^, ^31^, ^32^, ^33^ ^]^ redefining the gold standard for this class of point‐of‐care testing.

The enormous potential of PtNPs in cellular environment and *in vivo* applications requires accurate size control and optimal dispersion/stability in biological media, as such properties jointly govern the internalization process within cells, the catalytic performances per mass unit, and the labeling ability for proteins and antibodies. Moreover, surface coating needs to leave sufficient NP surface free for the catalytic processes, whilst imparting stability and further functionalization opportunities (such as bioconjugation).

Here, we harness the ideally suited properties of citrate‐coated PtNPs with sizes ranging from 3 to 20 nm to exploit for the first time their catalytic activity within cells to visualize the products of POD‐like reaction (i.e., the oxidation product of DAB) by TEM, and we prove that this has strong implications in the development of efficient probes for cellular imaging. We show that the pathway of proteins can be imaged both at low and high magnification with TEM by visualizing the electron‐dense osmiophilic reaction products obtained through the efficient catalytic properties of Pt nanozymes. These results may redefine the standard approach of metal enhancement currently in use, providing improved performances in terms of imaging capabilities.

## 2 Results and Discussion

### 2.1 Physico‐chemical and Catalytic Properties of Pt Nanozymes

**Figure 1:**
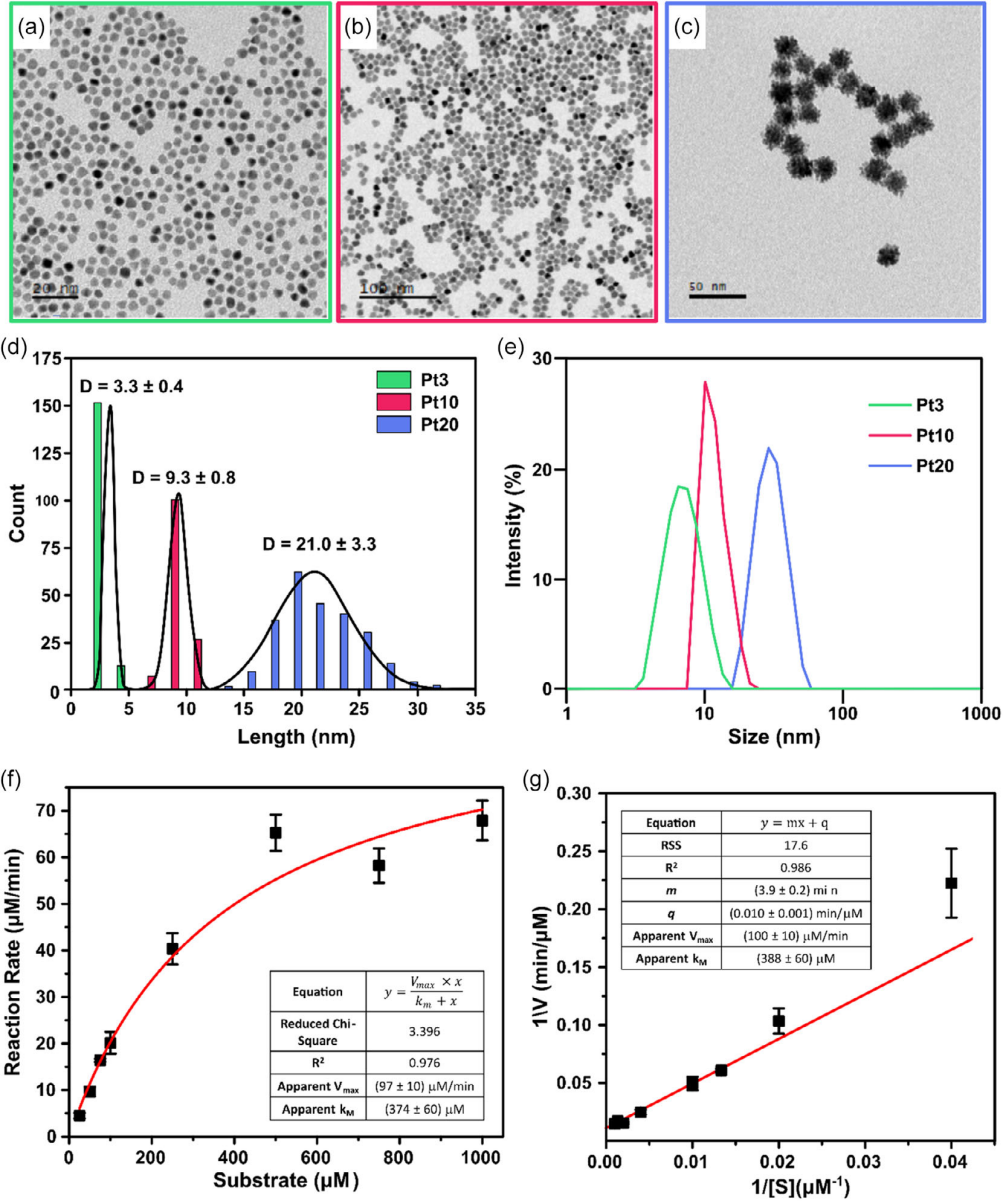
Representative TEM images of a) Pt3, b) Pt10, and c) Pt20, and d) NP size distribution of TEM and e) DLS analysis. *V*_max_ and apparent *k*_M_ of Pt3 calculated from the f) Michaelis–Menten equation and g) Linewaver–Burk plot. The two approaches provide similar results, with a *V*_max_ of about 100 μm min^−1^ and a *k*_M_ of 380 μm.

Purified citrate‐capped PtNPs, with sizes ranging from 3 to 20 nm and low polydispersity, were synthesized according to previously published protocol.^[^ ^10^ ^]^ Their physico‐chemical characterization was performed by TEM and dynamic light scattering (DLS) (**Figure** **1**). TEM imaging shows that PtNPs had narrow polydispersity in size and spherical shape, apart from the Pt20 particles characterized by flower‐like shape (Figure 1a–c). TEM size distribution plots (Figure 1d) indicate that NP size distributions were centered around 3 nm (Pt3), 10 nm (Pt10), and 20 nm (Pt20), while DLS displays stable and monodispersed particles in aqueous solution (Figure 1e). The size below 20 nm (and especially <5 nm) is pivotal to achieve abundant internalization in cells and high catalytic performances. Moreover, the choice of surface coating has important consequences on the material catalytic characteristics. Citrate molecules are ideally suited as they weakly bind to the surface and, hence, can be easily displaced (e.g., upon cellular uptake). More importantly, citrates only partially cover the surface, leaving the majority of catalytic sites easily accessible to the substrates (their “naked” surface area was calculated to be higher than 70% in case of comparable nanoparticles of 3 nm size).^[^ ^34^ ^]^ On the contrary, it has been observed that widely employed stabilizing agents, such as polymers, may have an adverse impact on numerous catalytic reactions as well as in the efficiency of cellular internalization.

The catalytic properties of citrate‐capped PtNPs were investigated using TMB as chromogenic substrate, extrapolating *V*_max_ and apparent *k*_M_ from Michaelis–Menten equation and Linewaver–Burker plot (Figure 1f,g). TMB (a methylated analogue of DAB) was chosen for its ability in aqueous environment to produce a clear, soluble blue product, facilitating accurate and reproducible measurements in UV‐Vis spectrophotometry compared to DAB that generates a brown precipitate upon oxidation, forming a polymerized deposit at the enzyme site.^[^ ^35^ ^]^ The calculated values of *V*_max_ (100 μm min^−1^) and apparent *k*_M_ (380 μm) show that PtNPs possess better catalytic features compared to other nanozymes previously reported in the literature and close to those of natural peroxidases.^[^ ^36^, ^37^, ^38^ ^]^ The specific activity of Pt3 was determined to be 133 U mg^−1^ (Figure S1A, Supporting Information). Moreover, the reaction kinetics of PtNPs of different sizes were compared both in solution and after deposition on a substrate (Figure S1B,C, Supporting Information), showing that 3 nm PtNPs perform slightly better than the other sizes thanks to their higher surface to volume ratio, which provides a larger number of active sites when the same mass of catalyst is used.

Notably, citrate coating imparts sufficient stability in biological media, a key parameter to deliver high performances within complex cellular environment. Moreover, the absence of sticky molecules, polymers, and surfactants coupled with the absence of toxic contaminants coming from the synthesis guarantees the biocompatibility of the nanomaterial.^[^ ^10^, ^14^ ^]^ Indeed, PtNPs are cytocompatible up to high concentration (100 μg mL^−1^),^[^ ^10^ ^]^ avoiding the onset of the cellular defense mechanisms, a major issue for biomedical and imaging applications. It is also important to stress that PtNPs are also highly resistant and do not degrade within the harsh endo‐lysosomal environment.^[^ ^10^, ^18^ ^]^ All these features render this nanomaterial ideal for applications in the biomedical and diagnostics fields, overcoming several limitations of current technologies.

### 2.2 Intracellular Catalytic Activity of Pt Nanozymes Imaged with Scanning TEM

**Figure 2:**
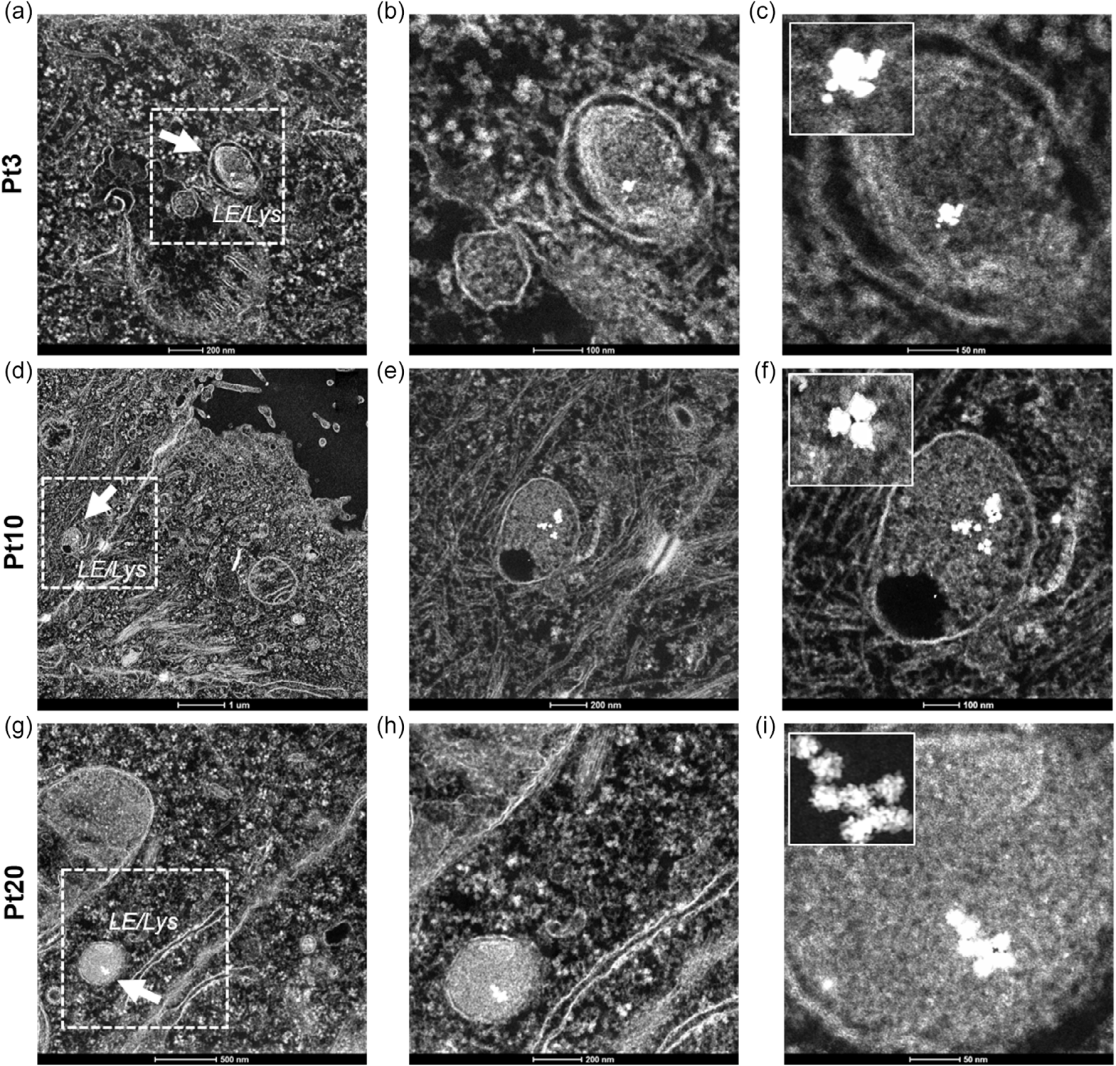
Low magnification HAADF STEM images of clusters of a) aggregated 3 nm, d) 10 nm and g) 20 PtNPs confined within late endosome/lysosome (LE/Lys) hybrids (white arrowheads). Close up views of the areas in the dashed line of b,c) Pt3, e,f) Pt10 and h,i) Pt20 are also reported.

The internalization and subcellular localization of Pt3, Pt10, and Pt20 after 24 h of exposure in HeLa cells were analyzed by high angular annular dark field (HAADF) scanning transmission electron microscopy (STEM). HAADF STEM images show that the NPs of all the three sizes were abundantly internalized by cells and were mainly present as clusters of aggregated particles confined within late endosome/lysosome (LE/Lys) hybrids (**Figure** **2**). The results highlight that the intracellular distribution of the NPs is predominantly influenced by the characteristics of their surface coating and the absence of targeting moieties rather than by the dimensions of the nanoparticles.

From a technical perspective, it is important to emphasize that at low magnifications (Figure 2a,d,g) NPs are poorly detectable despite their excellent contrast properties, because their nanometer size hinders their visualization within the complex biological environment. It is therefore necessary to employ a significant increase of magnification (Figure 2c,f,i) to resolve NP clusters within cells, even with an advanced and high‐resolution imaging technique like TEM. This casts severe limitations when systematic or quantitative analyses are required in nanomedicine applications, because it is necessary to explore many samples and many small fields of view, collecting a large number of different high‐resolution images, to gain statistically significant information.

**Scheme 1:**
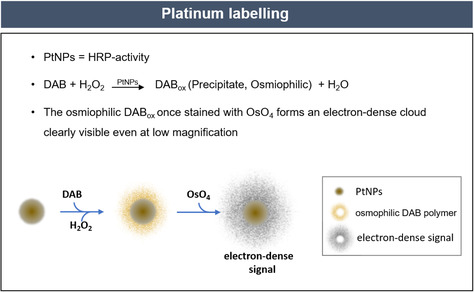
Representative scheme of the chemical process of signal amplification of PtNPs due to their intrinsic catalytic activity. PtNPs in solution behave as efficient POD‐like nanozymes. In the presence of DAB and hydrogen peroxide, PtNPs catalyze the oxidation of DAB, causing its polymerization in the area adjacent to the nanomaterial. This forms an osmiophilic precipitate, which is then stained with OsO_4_ that selectively binds to oxidized DAB and forms an electron‐dense cloud surrounding the PtNPs, highly visible in EM imaging at low magnification. Compared to HRPs, PtNPs offer the great advantage of high catalytic activity, stability, and resistance to harsh pH and temperature conditions.

In this framework, Pt nanozymes can provide the biomedical community with a powerful technical breakthrough. In aqueous solution, PtNPs behave as efficient POD‐like nanozymes, being able to promote the oxidation of chromogenic probes in presence of hydrogen peroxide. Here, we prove that it is possible to visualize the intracellular catalytic activity of PtNPs by TEM, using common catalytic substrates, such as DAB. DAB is a widely used chromogenic probe for peroxidase enzyme and is commonly employed as a staining agent in immune‐histological techniques. In the presence of DAB and hydrogen peroxide, PtNPs are able to catalyze the oxidation of DAB causing its polymerization in the area adjacent to the nanomaterial, forming an osmiophilic precipitate, which in turn has the potential to significantly amplify the staining of the NPs (**Scheme** **1**). Such precipitate becomes easily detectable thanks to the use of osmium tetroxide (OsO_4_) that has two main features: it selectively binds to oxidized DAB, and it is highly electron dense, thus creating high contrast within the organic matrix of the cells. This process leads to the formation of an electron dense cloud surrounding the PtNPs that is clearly visible in TEM imaging even at very low magnification (i.e., in large fields of view), overcoming major limitations of TEM imaging of small metallic nanoparticles in complex environment. Indeed, gold or silver nanoparticles, routinely in use for this task, pose challenges at low magnification in subcellular contexts or tissues, as they are particularly difficult to localize. To overcome such limitation, gold and silver enhancement is typically exploited but this technique is costly, time consuming, and prone to artefacts.

**Figure 3:**
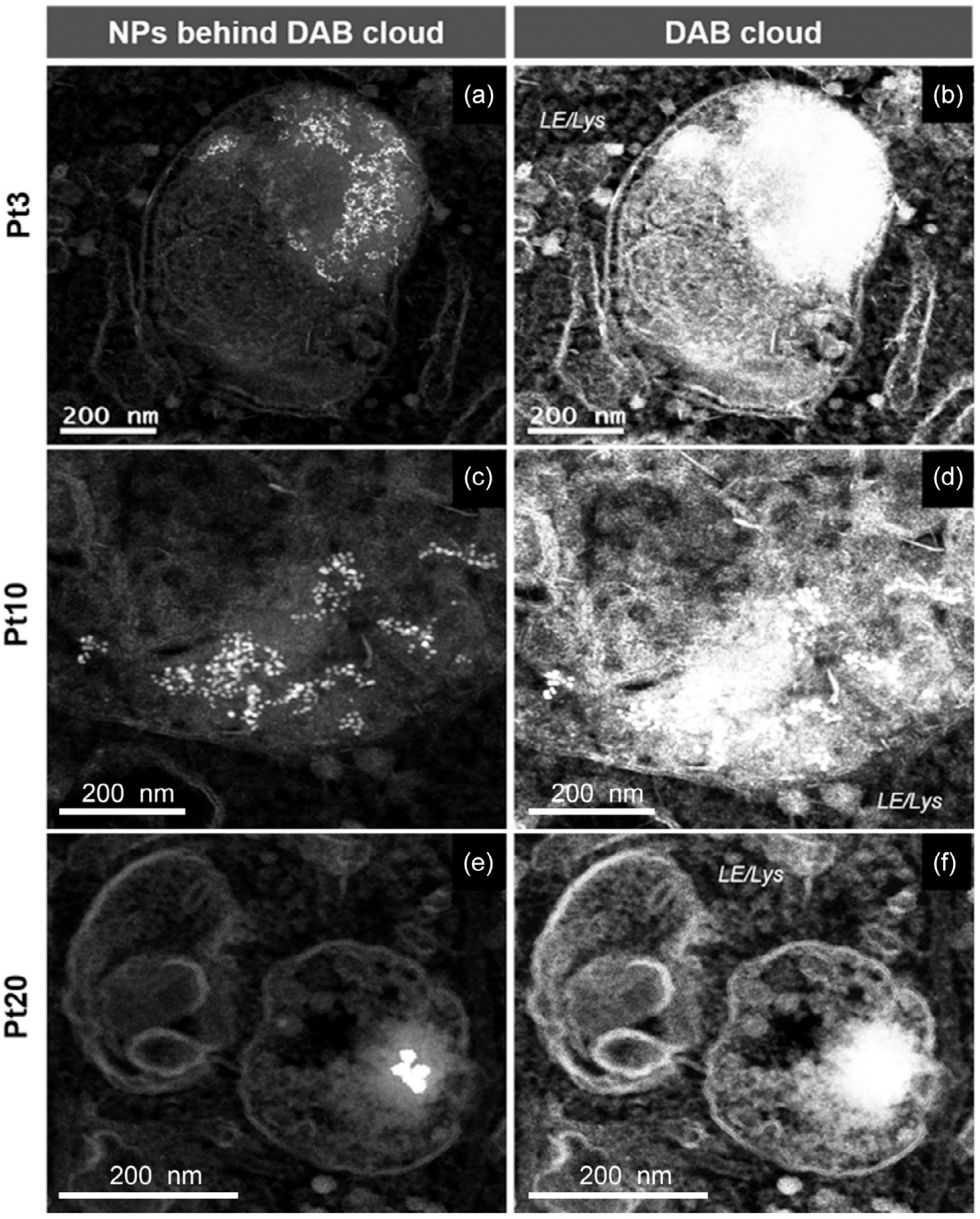
HAADF STEM images of PtNPs showing the amplification of the electron‐dense signal on NP surface by the addition of DAB and hydrogen peroxide, and the following staining with OsO_4_. a,c,e) NPs signal behind the DAB cloud showing single NPs in late endo/lysosomes and b,d,f) DAB cloud produced by the staining of oxidized DAB with OsO_4_.

PtNPs can provide a convenient alternative to these approaches thanks to their intrinsic catalytic activity. Interestingly, such enzymatic‐like functionality, proven in cell‐free environment, can be efficiently maintained within the complex cellular system. As shown in Figure 2, in the absence of DAB and hydrogen peroxide, NPs can be imaged within the endo‐lysosomes only at very high magnifications by TEM. In contrast, DAB and hydrogen peroxide addition amplifies the electron‐dense signal (**Figure** **3**) and, as a consequence, PtNPs become readily detectable in the context of the cellular ultrastructure at low magnification and across wide fields of view in the intricate intracellular milieu. Figure 3b,d,f proves a strong signal amplification, showing a lysosome completely filled with a DAB precipitate, stained with OsO_4_. Behind the cloud signal, PtNPs are easily detectable by scaling the micrograph normalized pixel intensity values (Figure 3a,c,e). The scaling does not change the magnification of the images but modifies the micrograph normalized pixel intensity values. Remarkably, as shown in Figure 3e,f, few particles (20 nm diameter) are sufficient to create a cloud of more than 100 nm in size.

**Figure 4:**
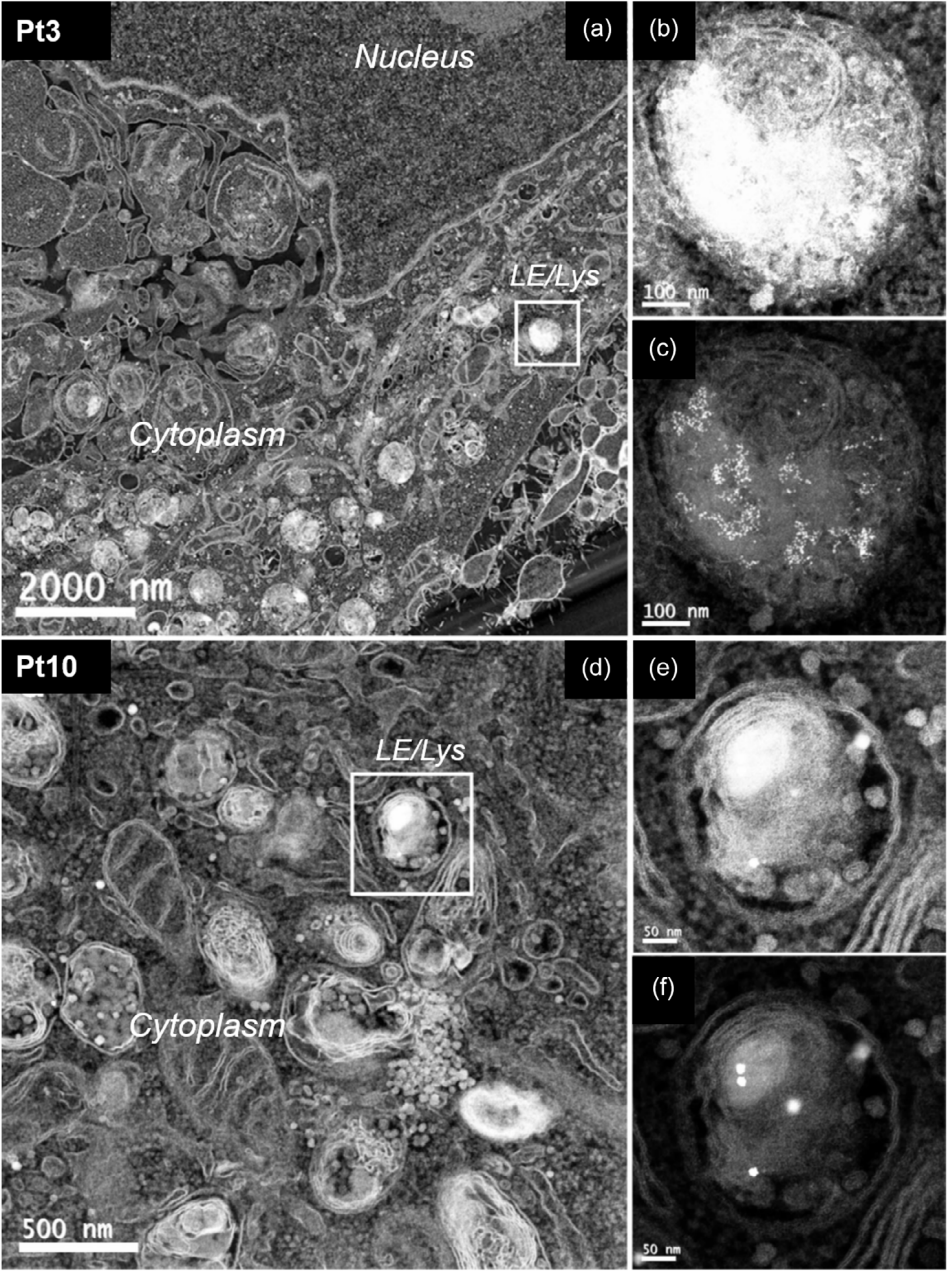
HAADF STEM images of amplified electron‐dense signal of PtNPs in a,d) endo‐lysosomes across wide fields of view, b,e) close up views of the DAB cloud, and c,f) NPs behind the cloud. Signal amplification obtained from the reaction with DAB makes NPs easily visible in the complex intracellular environment and in the context of the cellular ultrastructure using TEM, at relatively low magnifications and on very large fields of view, facilitating localization analysis in high throughput screenings.

In **Figure** **4**, images a and d clearly demonstrate that the electron‐dense cloud is evident at low magnification, whilst the panels b and c prove that PtNPs are responsible of this signal amplification and are visible behind the clouds (by scaling the micrograph normalized pixel intensity values). Images e and f further prove that few particles are sufficient to form a large cloud with at least an order of magnitude signal amplification (20 nm nanoparticles create a cloud of more than 200 nm diameter). Further images, in Figure S2, Supporting Information, prove the imaging capabilities of this nanosystem. Such catalytic signal amplification by Pt nanozymes opens new opportunities for localization and fate studies that require quick and high‐quality screening, like in the case of metal nanoparticles smaller than 10 nm in subcellular environments, in *ex vivo* tissues, or in environmental samples. Furthermore, as described in the following paragraph, PtNPs can also be employed as probes for immuno‐EM, demonstrating high potential for innovative imaging solutions.

### 2.3 Pt Nanozymes as Immuno‐EM Probes

**Figure 5:**
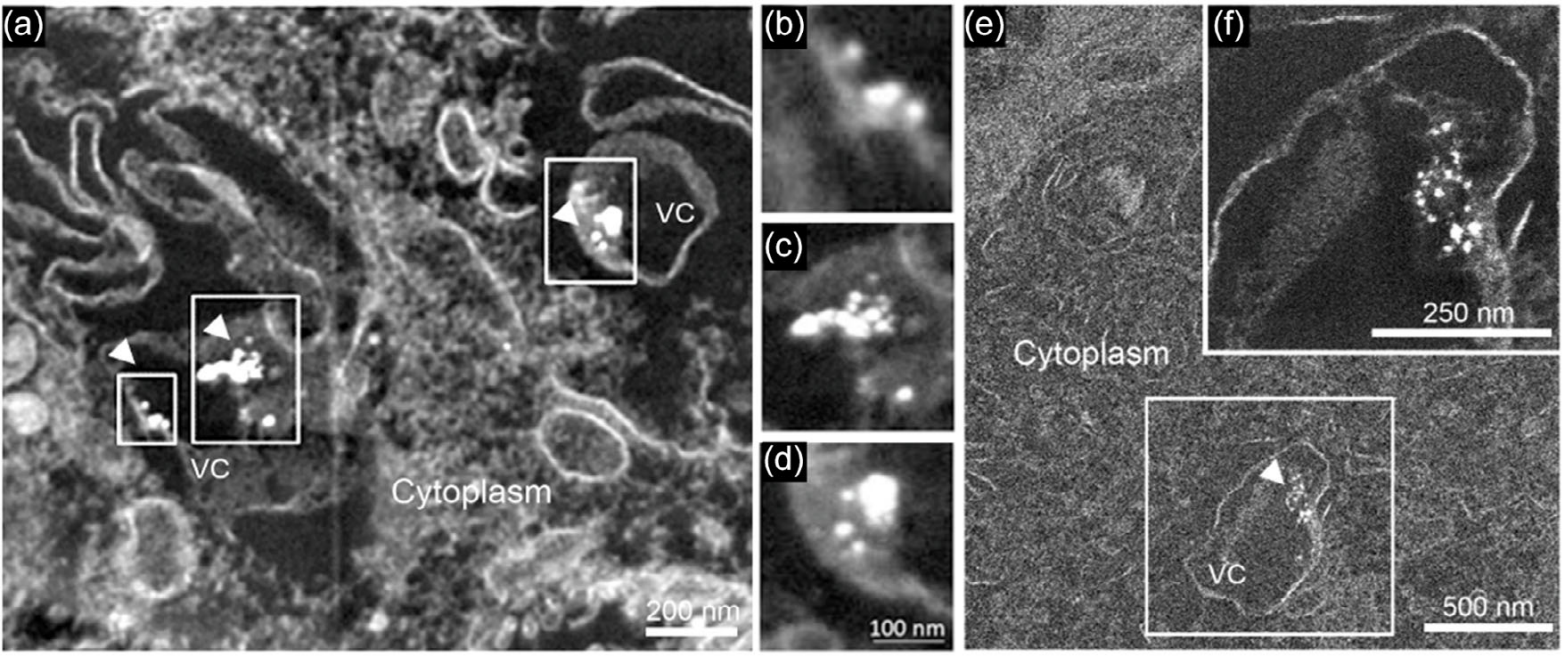
TEM images of LC3B subcellular localization visualized using a primary antibody against LC3B recognized by a Pt3‐conjugated secondary antibody. (a,e arrowheads) Electron‐dense spots corresponding to the complexes containing the NPs, polymerized DAB and OsO_4_ are confined in vesicular‐like compartments (VC). (b–d and f, respectively) A higher magnification of the white area is also shown.

In order to benefit from the easy localization of PtNPs in large fields of views in TEM imaging, we investigated the application of PtNPs as immuno‐EM probes to track the subcellular localization of endogenous molecules, with particular interest in proteins of proven difficult detection at the ultrastructural level with the common immuno‐EM labeling methods. Among these, the protein MAP1LC3B (microtubule associated protein 1 light chain 3B, LC3), a widely used marker of autophagic compartments at different stages of maturation, was chosen as a model target because of its well‐characterized cellular localization.^[^ ^39^ ^]^ During autophagosome maturation, LC3B protein is recruited on the membrane of autophagosomes while, after the fusion of autophagosomes with lysosomes, LC3B is internalized in auto‐phagolysosomes to be degraded.^[^ ^39^, ^40^ ^]^ Melanoma A375 cells, selected for their overexpression of the exogenous LC3B protein,^[^ ^41^ ^]^ were treated with a lysosomotropic compound^[^ ^42^ ^]^ to increase the numbers of intracellular auto‐phagolysosomes. Cells were processed for pre‐embedding immuno‐EM analysis. Pt3 particles were functionalized with a secondary antibody (AbII) able to bind the selected primary antibody (AbI) against LC3B. The size of 3 nm for PtNPs was selected as optimal because their dimension matches the “gold” standard of AuNPs, classically used in pre‐embedding immunogold techniques, favoring their ease penetration into the cell compartment, which is otherwise difficult for NPs of larger sizes. The functionalization of Pt3 with the secondary antibodies was performed by exploiting established techniques^[^ ^26^ ^]^ and characterized via agarose gel electrophoresis, which confirmed efficient Pt3‐AbII binding (Figure S3A, Supporting Information). Then, immuno‐EM was performed using Pt3‐AbII conjugate to stain LC3B protein in A375 cells, following standard pre‐embedding immuno‐gold protocols.^[^ ^43^ ^]^ In brief, after mild fixation, the cells were incubated with a rabbit polyclonal anti‐LC3B primary antibody and, after several washes, they were treated with the Pt3‐Ab II conjugate. For the PtNP development experiments, the following steps were added to the standard pre‐embedding protocol. After Pt3‐Ab II incubation, the cells were incubated in a buffer solution containing DAB and H_2_O_2_. Then, the cells were treated with osmium tetroxide (OsO_4_), stained with an aqueous uranyl acetate solution, dehydrated in an increasing alcohol series, and then embedded in epoxy resin. In the treated samples, the presence of electron‐dense spots was easily observed, corresponding to the complexes containing PtNPs, polymerized DAB, and OsO_4_. These spots are visible in the proximity of membranes delimiting vesicles filled by electron‐dense degraded cytoplasmic material. Concerning their ultrastructure, these labeled vesicles are compatible with phagolysosomal compartments^[^ ^44^ ^]^ (**Figure** **5**).

The specificity of the labeling of LC3B with Pt nanozyme probes in immuno‐EM experiments was evaluated semi‐quantitatively (Figure S3B, Supporting Information). Remarkably, in the treated samples, the Pt probe labeling was observed in correspondence of phagosomal compartments in the large majority of cases (94%) while, as expected for a non‐specific signal, in the negative controls Pt labeling was observed only in the 6% of the phagosomal structures.

Overall, we provided evidence that the high peroxidase‐like activity of PtNPs can be exploited to perform immune‐labeling of proteins for immuno‐EM, revealing the localization of the target protein at low magnifications with high sensitivity and specificity.

### 2.4 Pt Nanozymes as EM Probes of Protein Trafficking within Cells

**Figure 6:**
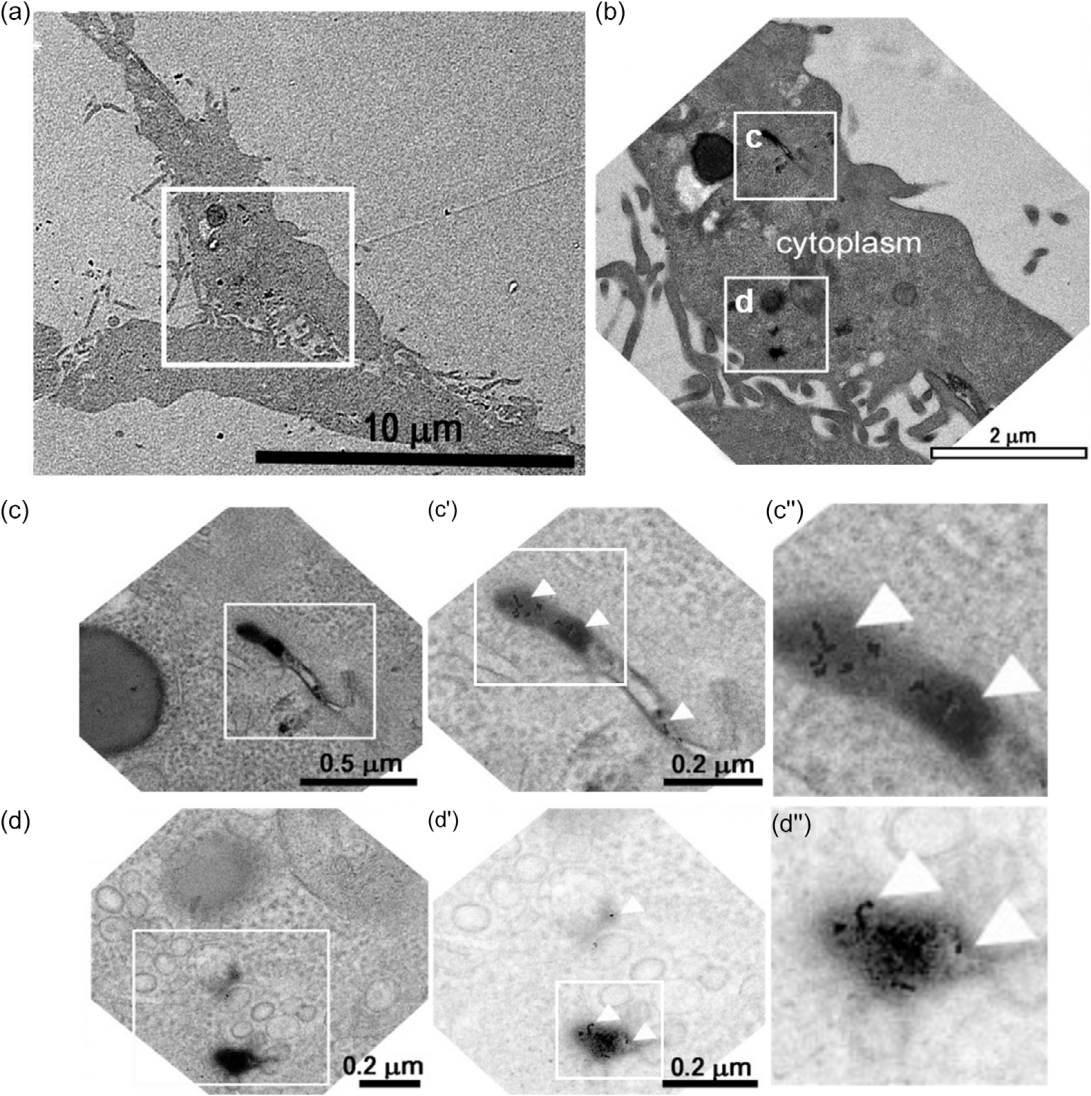
TEM images of transferrin conjugated‐PtNPs endocyted by cells within early endosomes as expected for transferrin, a) showing the presence of an electron‐dense signal visible at low magnification. b) At higher magnification NPs are detectable within the electron‐dense regions, c,d) highlighted in the white areas, further magnified in c′–c″ and d′–d″). Arrowheads show the NPs behind the DAB cloud.

The efficacy of PtNPs as EM probes has also been tested in TEM imaging of protein trafficking. As a model case, we used the pathway of transferrin (Tf), a major serum glycoprotein that transports iron into cells, which is widely employed as a marker to analyze trafficking processes within the endosomal cellular system. It is known that transferrin enters cells by receptor‐mediated endocytosis. Once in the endosomes, after iron dissociation, transferrin remains bound to its receptor and then is recycled back to the plasma membrane from primary endosomes.^[^ ^45^ ^]^ Therefore, this well‐established model is ideally suited to prove the potential of a bio‐conjugated structure composed of Pt3 and fluorescent transferrin (Alexa Fluor 594 conjugated transferrin, Tf_594_). EDC/NHS coupling chemistry was used to covalently bind the Pt3 with the fluorescent protein (Pt3@Tf_594_) (Figure S4A,B, Supporting Information). HeLa cells were incubated with Pt3@Tf_594_ and the conjugate localization was analyzed in TEM sections (**Figure** **6**). We observed that the staining of Pt probes presented the characteristic punctate pattern, consistent with a localization in membrane‐bound compartments. The amplification procedure revealed the presence of an intense electron‐dense signal recognizable even at low magnification (Figure 6a). TEM images at higher magnification showed the presence of individual Pt particles within the electron‐dense regions (Figure 6b–d″). Remarkably, the bioconjugated Pt probes were indeed present inside structures presenting the features of early endosomal tubules. We confirmed these results by confocal microscopy analyses. A clear colocalization of Pt3@Tf_594_ with the early endosome marker EEA1 (Figure S4Ca–e, Supporting Information) was observed, as expected for endocyted transferrin,^[^ ^46^ ^]^ rather than with lysosomes (Figure S4Cf–j, Supporting Information) where non‐functionalized PtNPs are typically localized.^[^ ^10^ ^]^

Hence, EM analyses coupled to confocal microscopy proved that the Pt probes are effective to visualize the trafficking of proteins within cells, opening interesting perspectives also in correlative light‐electron microscopy applications.

## 3 Conclusions

In this work, we have demonstrated the possibility to exploit Pt nanozymes as EM probes, for immuno‐EM as well as for the study of intracellular pathways of proteins. The use of Pt nanozyme probes offers multiple advantages over currently available techniques. PtNPs‐based EM probes overcome the need of silver or gold enhancement typically necessary for immunogold experiments. Classical AuNP probes, once inside the cells, are not readily detectable and need to be enhanced to be identified by TEM: the enhancement that occurs by nucleation from a solution of Ag or Au compounds in the presence of reducing agents is a multi‐step, time‐consuming, and complex procedure that can produce non‐specific signals and make results interpretation more challenging. On the contrary, the use of intrinsically catalytic PtNPs does not require the silver/gold enhancement step, with clear advantages in terms of experimental simplification, improved reliability, and reproducibility. Thanks to their strong signal enhancement, it is possible to recognize the Pt probes within the cells using TEM at relatively low magnifications and in very large fields of view. The amplification of the signal obtained from the reaction with DAB enables Pt probes recognition by TEM even in complex intracellular environments and in the context of cellular ultrastructures. Also, the intracellular catalytic activity of PtNPs would open up new possibilities for studying the cellular fate of metal nanoparticles smaller than 10 nm, which are difficult to observe with electron microscopy techniques in subcellular contexts. Another relevant advantage of this technique relies on the possibility to perform double EM immunolocalizations. The fate of different proteins could be visualized thanks to designed changes in the electron‐dense cloud. These variations can be easily obtained by tuning the catalytic activity of the NPs, for example, by tailoring their size, shape, surface area, and/or alloying different active materials.

## 4 Experimental Section

4.1

4.1.1

##### Cell Cultures

HeLa cells (human cervix epithelioid carcinoma cells, ECACC) were cultivated in DMEM (Sigma‐Aldrich) supplemented with 10% (v/v) FBS (Sigma‐Aldrich), 100 U per mL penicillin and 100 mg per mL streptomycin (Sigma‐Aldrich). Melanoma A375 cells (ATCC) overexpressing LC3^[^ ^41^ ^]^ were grown in DMEM high glucose (Sigma‐Aldrich) containing 4 mm L‐glutamine (Sigma‐Aldrich), 10% FBS (v/v), 0.5 mm sodium pyruvate (Sigma‐Aldrich) and 100 U per mL penicillin and 100 mg per mL streptomycin. The cells were incubated at 37 °C under a humidified atmosphere with 5% CO_2_.

##### Chemicals and Materials

All chemicals and reagents employed were of high technical grade, stored following vendor recommendations, and directly used with no further purification.

Boric acid (H_3_BO_3_, BioReagent, for molecular biology, suitable for cell culture, suitable for plant cell culture, ≥99.5%), L‐ascorbic acid (C_6_H_8_O_6_, suitable for cell culture, suitable for plant cell culture, ≥98%), Chloroplatinic acid hexahydrate (H_2_PtCl_6_·6H_2_O, BioXtra), citric acid (HOC(COOH)(CH_2_COOH)_2_, BioUltra, anhydrous, ≥99.5% (T)), phosphate buffered saline (tablet), sodium borohydride (H_4_BNa, granular, 99.99% trace metal basis), sodium citrate tribasic dihydrate (C_6_H_5_Na_3_O_7_·2H_2_O, BioUltra, for molecular biology, ≥99.5%), sodium tetraborate decahydrate (Na_2_B_4_O_7_·10H_2_O, ReagentPlus, ≥99.5%), Amicon Ultra‐4 Centrifugal Filter Unit (Ultracel‐3 regenerated cellulose membrane, 4 mL sample volume) were purchased from Merck (Sigma‐Aldrich).

Distilled, deionized water (Millipore, Milli‐Q system) was used for all solution preparation. For immune‐EM experiments, A375 cells were treated with 10 μm of the lysosomotropic compound 30^[^ ^42^ ^]^ for 24 h to enrich the intracellular number of autophagolysosomes.

For transferrin‐PtNPs conjugation, Transferrin from Human Serum, Alexa Fluor594 Conjugate was purchased by Thermo Fisher (Catalog number: T13343).

##### Platinum Nanoparticles Synthesis and Characterization

Citrate‐capped PtNPs, with sizes ranging from 3 to 20 nm and low polydispersity, were synthesized according to previously published protocols.^[^ ^10^ ^]^ The synthesis of Pt3 involves the reduction in water at high temperature of 0.5 m H_2_PtCl_6_, exploiting both reducing agents and capping agents (sodium borohydride, trisodium citrate, and citric acid). Both the syntheses of Pt10 and Pt20 are seed‐mediated in hot water starting from spherical seeds of 5 nm, and involving the reduction of 0.5 m H_2_PtCl_6_ exploiting the use of both reducing and capping agents (trisodium citrate and ascorbic acid) in different ratios.

The morphological and dimensional characterizations of PtNPs were performed by DLS (Malvern‐PANalytical), UV‐Vis spectrophotometry (Thermo Fisher NanoDrop, wavelength accuracy ±1 nm, absorbance accuracy 3% at 0.74 Abs @ 350 nm), and TEM (JEOL JEM‐1400Plus TEM, with LaB_6_ thermionic source and maximum acceleration voltage 120 kV). The nanoparticles’ size was determined by measuring at least 150 nanoparticles using ImageJ software (NIH).

##### Characterization of the POD‐Like Activity of PtNPs

The POD‐like activity of PtNPs was evaluated using TMB. DAB and TMB are both chromogens used in peroxidase‐based biochemical assays. While both produce measurable colored products, they differ significantly. DAB generates a brown precipitate upon oxidation, forming a polymerized deposit at the enzyme site, useful in microscopy but challenging for quantitative assays. In contrast, TMB yields a soluble blue product, simplifying spectrophotometric quantification.^[^ ^35^ ^]^

Michalis–Menten kinetics: 3 nm PtNPs were incubated in acetate buffer (10 mm pH 4.5), in the presence of 10 mm H_2_O_2_ varying the concentration of TMB from 0.025 to 1 mm. The absorbance spectrum of the solution was acquired after 1 min of incubation and the absorbance at 650 nm was converted in concentration of product formed using the extinction coefficient of oxidized TMB (39 000 m ^−1^ cm^−1^).

##### Pt3‐Ab II Conjugates Preparation and Characterization

Pt3 were functionalized with a secondary anti‐rabbit antibody (goat anti‐rabbit IgG (H + L), A‐21 429) in 5 mm borate buffer pH ≈ 8.6. After 1 h of incubation (300 rpm) at room temperature, the solution was washed using 3 K Amicon Ultra centrifugal filters, and then resuspended in 10 mm PBS pH = 7.4. The final concentration of PtNPs was determined by ICP‐OES analysis, while the amount of secondary antibody was quantified through MicroBCA assay. The effective conjugation was confirmed by agarose gel electrophoresis on 1% agarose gels using sodium boric acid (SB) buffer pH 8.5, comparing the conjugates with a sample of naked Pt3 (not conjugated).

##### STEM

HeLa cells were incubated for 24 h with Pt3, Pt10, and Pt20 nanoparticles and processed as previously reported.^[^ ^47^ ^]^ Briefly, after nanoparticle incubation, the cells were fixed for 1 h in 1.2% glutaraldehyde in 0.1 m sodium cacodylate buffer (pH 7.4). This fixation protocol is effective in inactivating peroxidases. Glutaraldehyde is a potent cross‐linking agent that inhibits enzymatic activities, including peroxidase activities. This property of glutaraldehyde is crucial for preventing non‐specific catalytic reactions such as DAB oxidation, thereby minimizing background staining and enhancing the specificity and clarity of immunohistochemical staining results.^[^ ^48^, ^49^ ^]^ Then the cells were incubated in 0.1 m NaCacodylate pH 7.4 + 0.3 mg mL^−1^ filtered DAB + 0.003% H_2_O_2_ (average 1.3 mm) until brown color develops. After several washes with cold H_2_O, the cells were post fixed in a buffer solution containing 1% osmium tetroxide and en blocstained with 1% uranyl acetate aqueous solution. The cells were then dehydrated in a graded ethanol series and embedded in epoxy resin (Epon 812, TAAB). Semi‐thin and thin sections of the embedded cell monolayer were cut with an ultramicrotome (UC6, Leica) equipped with a diamond knife (Diatome). The projection images were acquired in STEM with a HAADF detector, using a FEI Tecnai F20 TEM operating at 200 kV and equipped with a Schottky field‐emission gun.

##### Immuno‐EM for the Staining of LC3B with Pt3‐Ab II

A375 cells were fixed with PFA 4% and 0.1% glutaraldehyde in PBS 0.1 m, then permeabilized with saponine 0.02% in blocking solution (0.1 m TRIS buffer, 10% BSA), extensively washed in washing solution (0.1 m TRIS 1% BSA), and incubated overnight at 4 °C in rabbit polyclonal anti‐LC3B primary antibody (LC3B Antibody, cat no. 2775, Cell Signaling) in incubation buffer (10% BSA, 0.004% saponine in TRIS 0.1 m). After several washes in washing buffer, the cells were incubated with the Pt3‐conjugated anti‐rabbit Ab II in incubation buffer for 4 h at room temperature. The cells were then toughly washed in washing buffer, and then post‐fixed for 10 min in 1% glutaraldehyde in PB 0.1 m. Then, the cells were incubated in 0.1 m PB buffer (pH 7.4) containing 0.3 mg mL^−1^ filtered DAB and 0.003% H_2_O_2_ until brown color develops. After several washes with cold H_2_O, the cells were post fixed in 1% osmium tetroxide in MQ H_2_O, and en bloc stained with 1% uranyl acetate aqueous solution. The cells were then dehydrated in a graded ethanol series and embedded in epoxy resin (Epon 812, TAAB).

##### Preparation of Fluorescent Transferrin‐Conjugated PtNPs and CLSM

Transferrin (Tf) conjugation with thiol‐coated Pt3 was carried out by a covalent coupling of the carboxylic groups of the MPA layer with the primary amines of transferrin via carbodiimide chemistry. As crosslinking agents, N‐(3‐dimethylaminopropyl)‐N′‐ethylcarbodiimide hydrochloride (EDC) was used to mediate the formation of peptide linkages between Tf and MPA, and N‐hydroxysuccinimide (NHS) for improving the reaction rate and efficiency. Activation of Pt3 was carried out using EDC and NHS as follows: 15 μL of a solution 30 mg mL^−^^1^ of EDC (Sigma‐Aldrich, BioXtra) and 15 μL of a solution 40 mg mL^−^^1^ of NHS (Sigma‐Aldrich) were added to 1 mL of a solution 0.3 mg mL^−^^1^ of MPA/MPSA Pt3. The reaction mixture was gently shaken for 5 min at room temperature. The excess of EDC/NHS was immediately removed from the NP solution by centrifugation at 15 000 rcf for 30 min. Afterwards, the supernatant was collected in another vial, the pellet was resuspended in MilliQ water and both the solutions were centrifuged again. This washing procedure was repeated two more times. The NP solution was then resuspended into 1 mL of PBS. Tf‐conjugated PtNPs (Pt3@Tf_594_) were obtained by adding 20 μL of a solution 2 mg mL^−^^1^ of Tf_594_ (T13343, Thermo Fisher Scientific) to 1 mL of EDC/NHS activated NPs. The solution was gently shaken and incubated for 2 h at room temperature. The resultant Pt3@Tf_594_ was washed twice with PBS at 15 000 rcf for 30 min to remove unconjugated Tf_594_. HeLa cells were incubated with Pt3@Tf_594_ for 1 h and then stained with EEA1 primary monoclonal antibody (MA5‐31 575, Thermo Fisher Scientific) followed by a Goat anti‐Mouse IgG (H + L) secondary antibody Alexa Fluor 488 (A28175, Thermo Fisher Scientific) after fixation with 4% PFA or treated with LysoTracker Green DND‐26 (L7526, Thermo Fisher Scientific) to track early endosomes or lysosomes, respectively. CLSM was performed using a Leica TCS SP8 confocal microscope (Leica Microsystems, GmbH, Germany) equipped with a 63×oil‐immersion objective (HC PL APO CS2 63 × 1.40 OIL, Leica Germany). Imaging was performed using a white light laser (470–670 nm) and exciting Alexa Fluor 488 and LysoTracker Green DND‐26 at 488 nm, and Alexa Fluor 594 at 594 nm. Fluorescent emission was detected in the spectral window between 500 and 550 nm (Alexa Fluor 488 and LysoTracker Green DND‐26 emission), and 580–680 (Alexa Fluor 594 emission) by a GaAsP hybrid photodetector (Leica HyD).

## Conflict of Interest

The authors declare no conflict of interest.

## Supporting information

Supplementary Material

## Data Availability

The data that support the findings of this study are available from the corresponding author upon reasonable request.
