# Supplementary material for: Platinum Nanozyme Probes for Cellular Imaging by Electron Microscopy
Source: Small Sci. 2024 Jun 9;4(9):2400085. doi: 10.1002/smsc.202400085 (PMC11935043; doi:10.1002/smsc.202400085)
Supplement: Supplementary file 1 — Supplementary Material [file SMSC-4-2400085-s001.pdf]

Supplementary Information

Platinum nanozyme probes for cellular imaging by electron microscopy

E. De Luca[a,b,c], D. Pedone[a], A. Scarsi[a], R. Marotta[d], F. Catalano[d], D. Debellis[d], Lorenzo Cursi[a], B. Grimaldi[e], M. Moglianetti[a,f*] and P.P. Pompa[a*]

[a] Nanobiointeractions&Nanodiagnostics, Istituto Italiano di Tecnologia, via Morego 30, 16163 Genova, Italy

[b] CNR NANOTEC – Institute of Nanotechnology, c/o Campus Ecotekne, Via Monteroni, 73100 Lecce, Italy

[c] Istituto Italiano di Tecnologia, Center for Biomolecular Nanotechnologies, Via Barsanti – 73010 Arnesano, Lecce, Italy

[d] Electron Microscopy Laboratory, Nanochemistry Department, Istituto Italiano di Tecnologia, Via Morego 30-16163, Genova, Italy

[e] Molecular Medicine Research Line, Istituto Italiano di Tecnologia (IIT), Genoa, 16163, Italy

[f] Center for Cultural Heritage Technology (CCHT) Istituto Italiano di Tecnologia (IIT) Via Torino 155, Venezia 30172, Italy

*Corresponding authors

e-mail: pierpaolo.pompa@iit.it

e-mail: mauro.moglianetti@iit.it

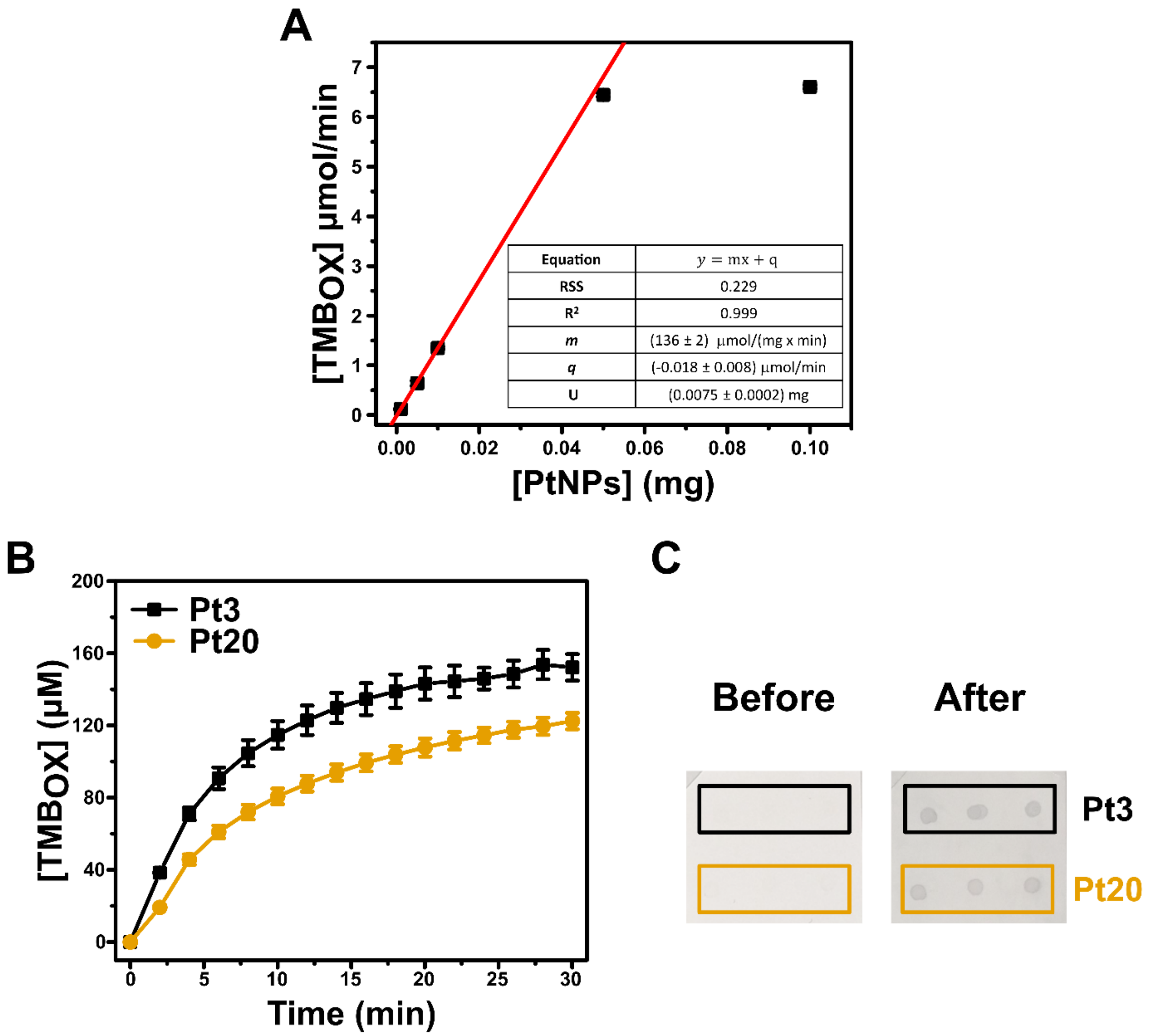

Figure S1. (A) Plot of TMB_{OX} formed in unit of time *vs.* mass of nanozyme, used to calculate the specific activity of Pt3 towards TMB. 1U of Pt3 (0.0075 mg) can oxidize 1 mmol of TMB per minute in the presence of H_2O_2 10 mM, in Acetate 10 mM pH 4.5 and 25 °C. **(B)** Comparison of POD-like activity between Pt3 and Pt20 in solution with TMB and **(C)** after deposition on Nylon membrane with DAB. The NPs were normalized by mass of catalyst (0.05 ppm in solution and 1 µL of a 5 ppm dispersion spotted on the substrate).

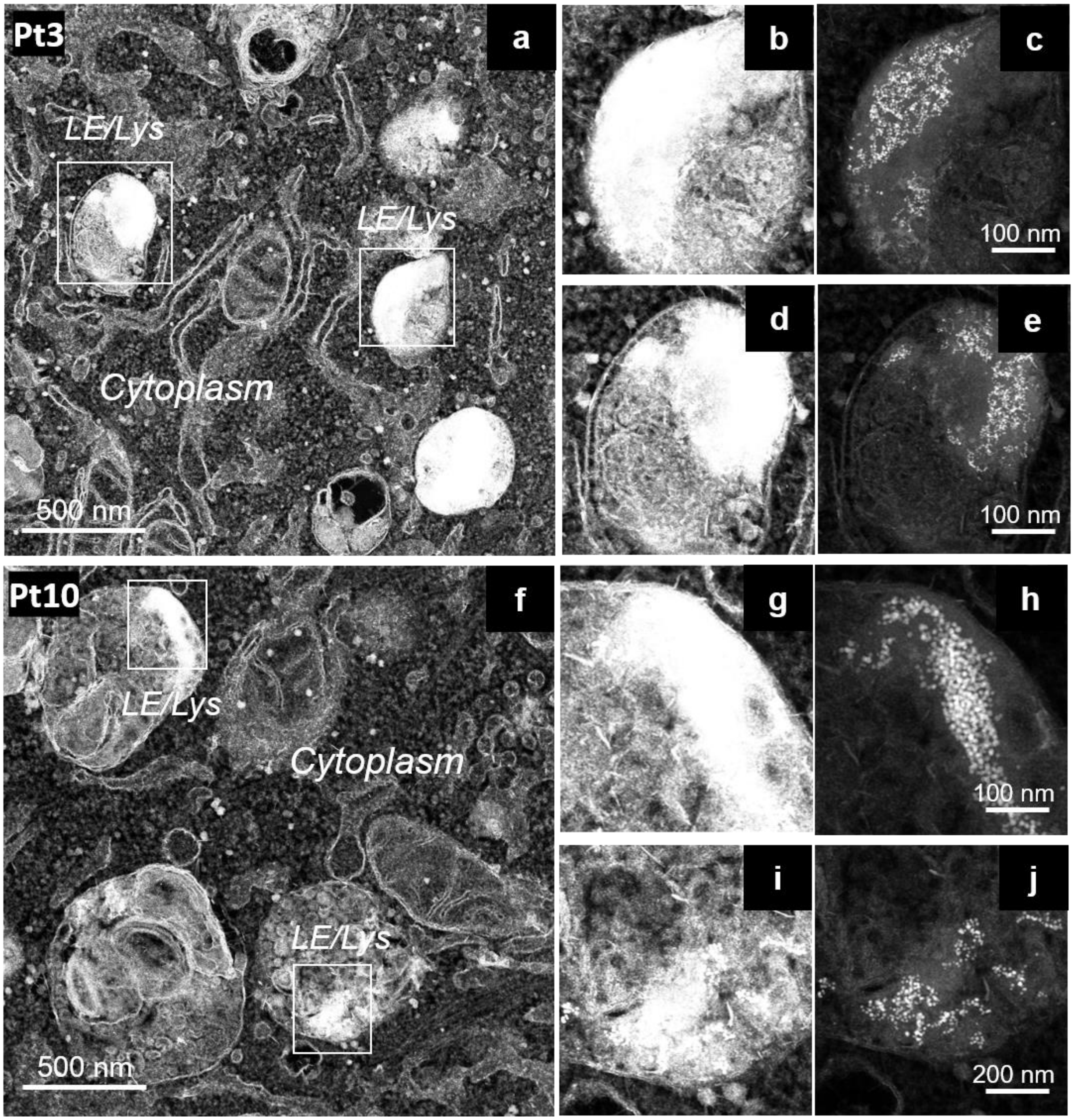

Figure S2. HAADF STEM images of amplified electron-dense signal of PtNPs in endolysosomes across wide fields of view (a, f), close up views of the DAB cloud (b, d, g, i) and NPs behind the cloud (c, e, h, j). Signal amplification obtained from the reaction with DAB makes NPs easily visible at relatively low magnifications and in large fields of view using TEM.

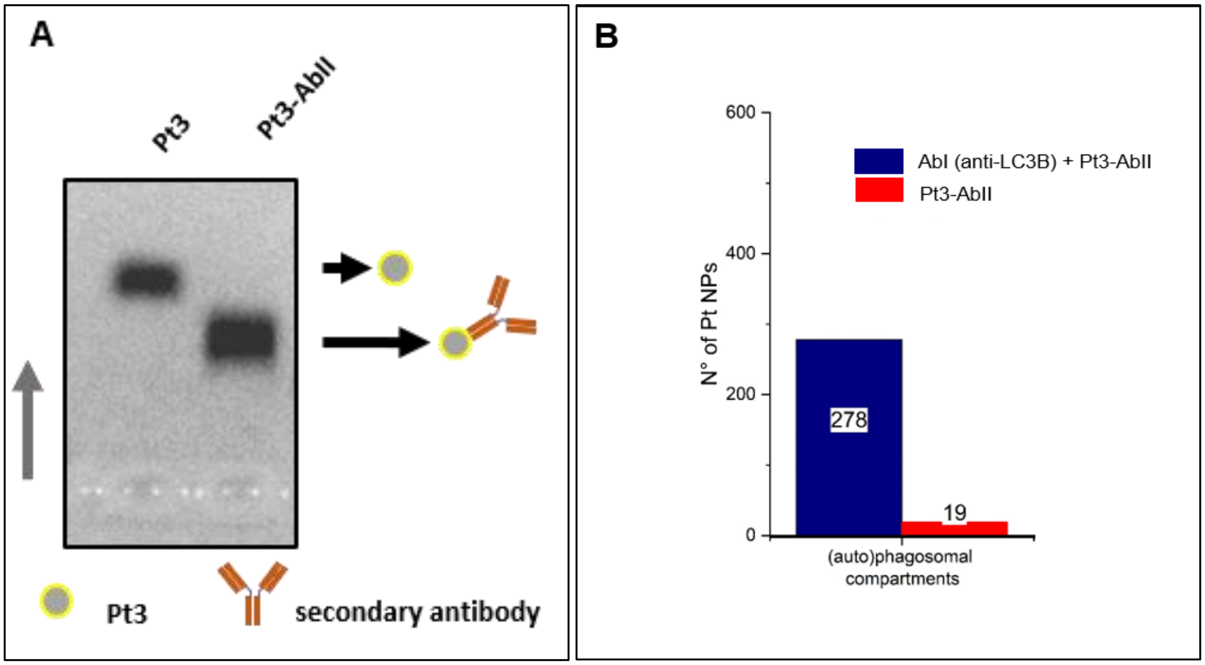

Figure S3. (A) Gel electrophoresis of unconjugated Pt3 and Pt3 functionalized with the secondary antibody. AbII-functionalized PtNPs (Pt3-AbII) show retarded electrophoretic mobility (1% agarose) compared to control PtNPs (Pt3). Grey arrow marks the mobility direction of NPs within the agarose gel. **(B)** Semi-quantitative analysis of the specificity of the labelling of LC3B with nanozyme probes, showing that Pt probe signal was observed in the phagosomal compartments in 94% of cases (blue column) against the 6% of signal in the negative controls (red column).

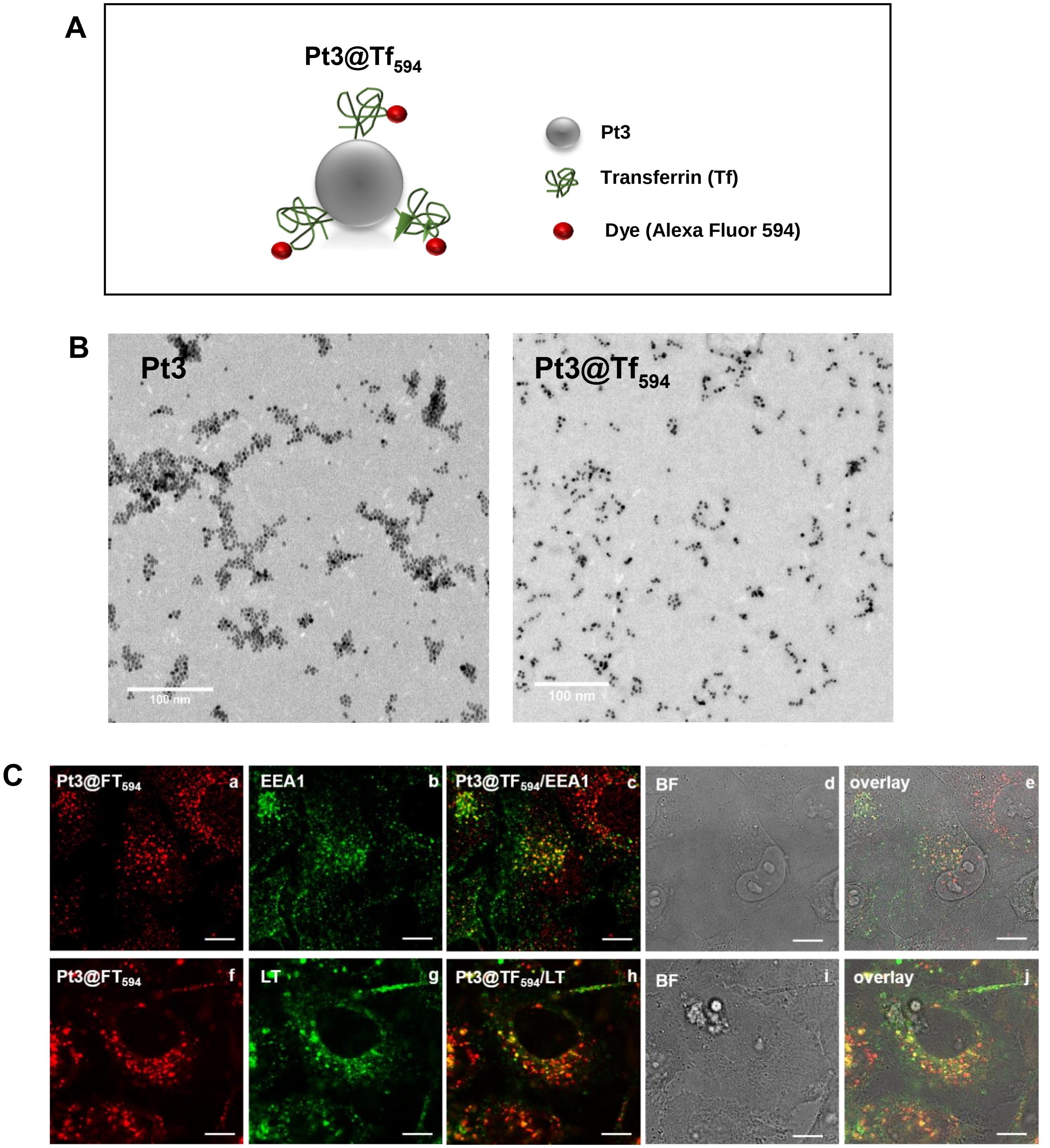

Figure S4. **(A)** Schematic representation of NP functionalization design. **(B)** TEM images of naked Pt3 and transferrin conjugated Pt3, Pt3@Tf594. **(C)** CLSM of HeLa cells incubated for 1h with Pt3@Tf594 and stained with EEA1 antibody (a-e) and Lysotracker Green (LT) (f-j) to follow the intracellular transferrin localization. BF: brightfield. Scale bar: 5 μm.
